# Biogeographic variation in the microbiome of the ecologically important sponge, *Carteriospongia foliascens*

**DOI:** 10.7717/peerj.1435

**Published:** 2015-12-17

**Authors:** Heidi M. Luter, Stefanie Widder, Emmanuelle S. Botté, Muhammad Abdul Wahab, Stephen Whalan, Lucas Moitinho-Silva, Torsten Thomas, Nicole S. Webster

**Affiliations:** 1 NAMRA and the Research Institute for the Environment & Livelihoods, Charles Darwin University, Darwin, Northern Territory, Australia; 2 CUBE, Department of Microbiology and Ecosystem Science, University of Vienna, Vienna, Austria; 3 Australian Institute of Marine Science, Townsville, Queensland, Australia; 4 Australian Institute of Marine Science, Crawley Western Australia, Australia; 5 Marine Ecology Research Centre, School of Environment, Science and Engineering,Southern Cross University, Lismore, New South Wales, Australia; 6 Centre for Marine Bio-Innovation and School of Biotechnology and Biomolecular Sciences,University of New South Wales, Sydney, New South Wales, Australia

**Keywords:** Sponge-associated microbial community, Carteriospongia foliascens, Co-occurrence networks (NWs), Biogeography

## Abstract

Sponges are well known for hosting dense and diverse microbial communities, but how these associations vary with biogeography and environment is less clear. Here we compared the microbiome of an ecologically important sponge species, *Carteriospongia foliascens*, over a large geographic area and identified environmental factors likely responsible for driving microbial community differences between inshore and offshore locations using co-occurrence networks (NWs). The microbiome of *C. foliascens* exhibited exceptionally high microbial richness, with more than 9,000 OTUs identified at 97% sequence similarity. A large biogeographic signal was evident at the OTU level despite similar phyla level diversity being observed across all geographic locations. The *C. foliascens* bacterial community was primarily comprised of *Gammaproteobacteria* (34.2% ± 3.4%) and *Cyanobacteria* (32.2% ± 3.5%), with lower abundances of *Alphaproteobacteria*, *Bacteroidetes*, unidentified *Proteobacteria*, *Actinobacteria*, *Acidobacteria* and *Deltaproteobacteria*. Co-occurrence NWs revealed a consistent increase in the proportion of *Cyanobacteria* over *Bacteroidetes* between turbid inshore and oligotrophic offshore locations, suggesting that the specialist microbiome of *C. foliascens* is driven by environmental factors.

## Introduction

Sponges are a diverse and significant component of benthic habitats worldwide (Bell et al., 2013), contributing to benthic-pelagic coupling through their filtering capacity (Reiswig, 1974), undertaking reef bioerosion and consolidation (Bell, 2008) and making a major contribution to recycling nutrients and energy for other reef organisms (De Goeij et al., 2013). On the Great Barrier Reef (GBR), phototrophic Dictyoceratid sponges are common and conspicuous taxa (Wilkinson & Cheshire, 1989; Duckworth et al., 2008), with species of *Carteriospongia*, *Phyllospongia* and *Strepsichordaia* comprising up to 80% of total sponge biomass on mid- and outer-shelf reefs (Wilkinson, 1988). These species provide habitats for a wide range of reef taxa and contribute to primary production via their productive symbioses with cyanobacteria (Wilkinson, 1983; Webster et al., 2013).

Sponges are known to host dense and diverse microbial communities (see Taylor et al., 2007; Webster et al., 2010; Lee et al., 2011; Schmitt, Hentschel & Taylor, 2012 and references cited within), yet little is known about how the associations vary with biogeography and environment. The most extensive biogeographical study to date examined 32 sponge species from eight geographic locations revealing a minimal core bacterial community and a large species-specific community (Schmitt, et al., 2012), a pattern subsequently confirmed in other species (Schmitt, Hentschel & Taylor, 2012). In addition, a recent study comparing two distinct color morphs of *Petrosia ficiformis* reported that biogeography, rather than cyanobacterial symbionts, was responsible for the variability observed in the microbial community between color morphs (Burgsdorf et al., 2014). Most experimental studies assessing how sponge microbial communities respond to different environmental conditions have demonstrated host-specific microbial responses to temperature, nutrients and sediments (Webster et al., 2011; Luter, Whalan & Webster, 2012; Simister et al., 2012a; Simister et al., 2012b; Fan et al., 2013; Pita et al., 2013; Luter, Gibb & Webster, 2014). Responses from these types of environmental conditions range from sponges maintaining highly conserved microbial communities, irrespective of ambient conditions, through to highly sensitive communities that shift composition and function in response to the changing environment. Similarly, in the few studies that have undertaken *in situ* analysis of microbial communities in sponges under varying irradiance or *p*CO_2_ levels, different species hosted either stable or variable microbial communities in the face of the altered environmental conditions (Morrow et al., 2014; Freeman et al., 2015).

Next generation sequencing technologies have greatly expanded our understanding of microbial community composition in a wide range of different systems (Lynch & Neufeld, 2015). Moreover, 16S rRNA gene datasets have also been used to infer correlation networks to shed light on possible interactions between microbial species, especially within complex communities. These statistical co-occurrence patterns can be used as a filter for functionality (in the case of ecological interactions) and offer insights into canalizing environmental conditions (e.g., environmental gradients) that explain the co-presence of two taxa, which is of particular interest for mechanistically-driven studies (Berry & Widder, 2014; Widder et al., 2014).

This study aimed to determine biogeographic variation in the microbial community of the ecologically important sponge, *Carteriospongia foliascens*, and subsequently use community co-occurrence networks to identify potential shifts in key taxa between inshore and offshore environments. Increased agriculture of coastal Australian land since European settlement (Kroon, Kuhnert & Henderson, 2012), and associated land run-off to coastal waters, has been a significant contributor to turbidity of the inshore GBR(Furnas, 2003). As previous sponge symbiosis studies have demonstrated reduced chlorophyll a concentrations under low light conditions (Becerro & Paul, 2004; Erwin & Thacker, 2008), we hypothesized that site characteristics reflecting light levels would drive differences in sponge microbial communities between inshore and offshore environments. In particular, *Cyanobacteria* were expected to be adversely affected by the increased turbidity and reduced light levels of inshore environments, potentially being replaced by taxa that are more efficient consumers of dissolved organic matter (DOM) (Pereira, 2010).

## Materials & Methods

### Sample collections

Individuals of *C. foliascens* were collected from multiple locations around tropical Eastern and Western Australia as part of a wider phylogenetic study on foliose keratose sponges, see (Abdul Wahab et al., 2014b). The present study focused on 72 individuals identified as Evolutionary Significant Unit (ESU) I (Abdul Wahab et al., 2014b) from the following locations: Davies Reef (*n* = 15), Green Island (*n* = 13), Fantome Island (*n* = 14), Orpheus Island (*n* = 15), Torres Strait (Masig Island, *n* = 7), Scott Reef (*n* = 3) and the Dampier Archipelago (*n* = 5). Tissue samples were preserved in 100% ethanol and stored at −20 °C prior to molecular analysis. Samples were collected under the Great Barrier Reef Marine Park Authority Permit #G12/35236.1.

### DNA extraction, sequencing & processing

Genomic DNA was extracted using the PowerSoil htp 96-well DNA Isolation Kit (MoBio Laboratories, Inc.), following the manufacturer’s protocol. The 16S rRNA genes were amplified by PCR and sequenced as part of the Earth Microbiome Project (EMP) (Gilbert, Jansson & Knight, 2014) on the Illumina platform using the bacterial primers 515F/860R and standard protocols (Caporaso et al., 2012).

Quality-filtered, demultiplexed fastq sequences provided by the EMP were processed using Mothur v.1.31.2 (Schloss et al., 2009). Sequences were filtered using the following parameters: average quality score = 30, window size = 5 bases, maximum ambiguity = 0 and maximum number of homopolymers = 8, and trimmed to 100 bp. Unique sequences were aligned against a trimmed SILVA database (v102, trimmed to the V4 region) and chimeric sequences identified by UCHIME (Edgar et al., 2011) were removed. Classified sequences were grouped into operational taxonomic units (OTU) at 97% sequence similarity using the furthest neighbor clustering method. Representative sequences were classified based on the SILVA database, using a minimum cutoff of 60%. Singletons, i.e., OTUs formed by one sequence across all samples, were removed. Processed sequences and meta-data are available via the following portal (http://qiita.microbio.me/) under study number 1740. Rank abundance plots were created using BiodiversityR 2.5-2 (Kindt & Coe, 2005) and diversity metrics using rarefied data (*n* = 7,370 sequences) were created using vegan 2.3-0 (Oksanen et al., 2015), both packages in R.

### Data analysis

Principal coordinate analysis (PCO) was used to visually compare *C*. *foliascens* communities and PERMANOVA, using 9,999 permutations, was used to test differences in community structure between the different geographic locations. PRIMER/PERMANOVA’s Mantel-type test, RELATE, was used to compare similarity matrices and hierarchal clustering of sample locations was achieved using CLUSTER analysis. Similarity Percentage (SIMPER) analysis was used to determine the OTUs that contribute to the differences in community structure between Eastern Australian samples from locations classified as either inshore (Green, Fantome and Orpheus Islands) or offshore (Davies Reef). OTUs from the SIMPER analysis were visualized using Cytoscape v3.2.1 (www.cytoscape.org) (Shannon et al., 2003). All statistical analyses were based on Bray-Curtis distances of standardized (by sample) square root transformed data and performed using PRIMER 6/PERMANOVA+ v1.0.2 (Plymouth, UK).

Sequences from the 30 most abundant OTUs were aligned using the SINA web aligner (Pruesse, Peplies & Glöckner, 2012). Maximum likelihood analysis and tree construction were performed in MEGA v6.06 (Tamura et al., 2013), using the Kimura 2-parameter model with a gamma distribution. Tree reliability was tested by computing 1,000 bootstrap replicates starting with a neighbor-joining tree and using the nearest-neighbor interchange (NNI) tree search option.

### Co-occurrence networks

OTU abundance data were split into offshore and inshore samples and further processed independently. We inferred co-occurrence networks (NWs) using the sparCC algorithm (Friedman & Alm, 2012) and considered a robust co-occurrence event, if sparCC correlation >|0.6| and *p*-value ≤0.03. Subsequently, we performed a False Discovered Rate (FDR) correction according to Benjamini & Hochberg, (1995). To determine the fragmentation pattern of the co-occurrence community, we further simplified the NWs and removed anti-correlation edges and nodes with degree *k* > 15, which are likely artifacts of the pairwise correlation algorithms. Fragmentation, *f*, was calculated according to *f* = log(*CL*)∕log(*N*), where *CL* is the number of topological clusters in the graph and, *N*, the number of nodes (Widder et al., 2014). NWs were visualized in Cytoscape (Fig. S2).

For evaluating a shift in predominant taxa between geographical locations, we used the set of all OTUs present in either the inshore NW or the offshore NW. The ratio between *Cyanobacteria*/*Bacteroidetes* within this set was calculated using the mean relative abundances of taxa belonging to either of the two phyla. Inference and analyses were performed in R (R Core Team, 2015).

## Results and Discussion

### Overall community composition

**Figure 1:**
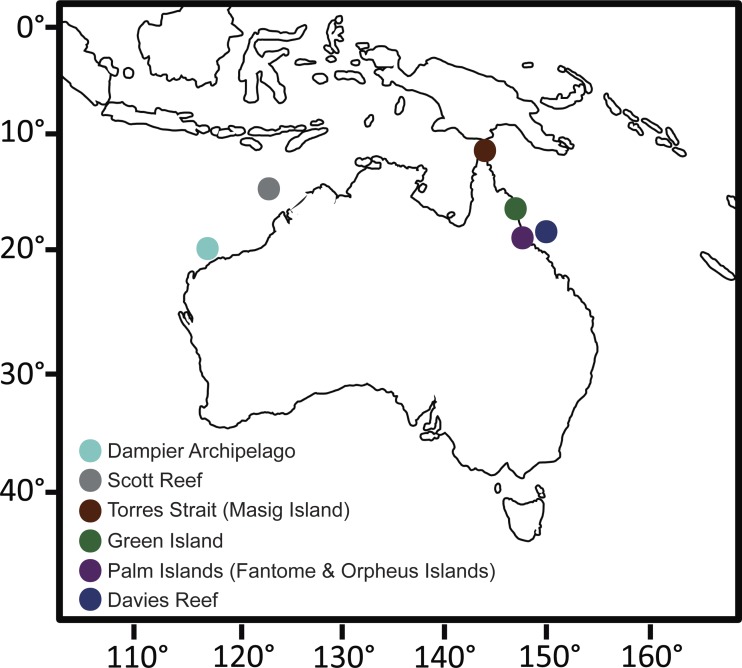
Collection site map. Map of Australia showing the sampling sites for this study. Due to the close proximity of Fantome and Orpheus Island (<10 km apart), both islands are represented by a single Palm Islands symbol. GPS coordinates for each site can be found in Table S1.

As part of a global sponge microbiome initiative, forming part of the EMP, the microbial communities of 72 *C. foliascens* individuals collected from multiple locations around tropical Eastern and Western Australia were sequenced (Fig. 1 and Table S1). A total of 9,401 OTUs, spanning 15 bacterial phyla, were identified across all individuals at 97% sequence similarity. These OTUs comprised 1,382,146 sequences, with an average of 19,196.47 (±7,972.81 1 S.D.) per sample. Representative sequences averaged 98.98 bp (±0.17 1 S.D.) in length. *C. foliascens* hosts exceptional bacterial richness, exceeding the number of unique OTUs reported for 20 other sponge species also sequenced as part of the EMP (Easson & Thacker, 2014). However, it is important to note that direct comparison of OTU richness between studies is often confounded by biases introduced in the laboratory and computational processing steps. For instance, whilst *C. foliascens* has exceptionally high OTU level diversity compared to other reported sponge species, Easson and colleagues used a minimum threshold of 500 reads (compared to a threshold of 2 reads in the current study), likely contributing to the higher apparent diversity in*C. foliascens*.

**Figure 2:**
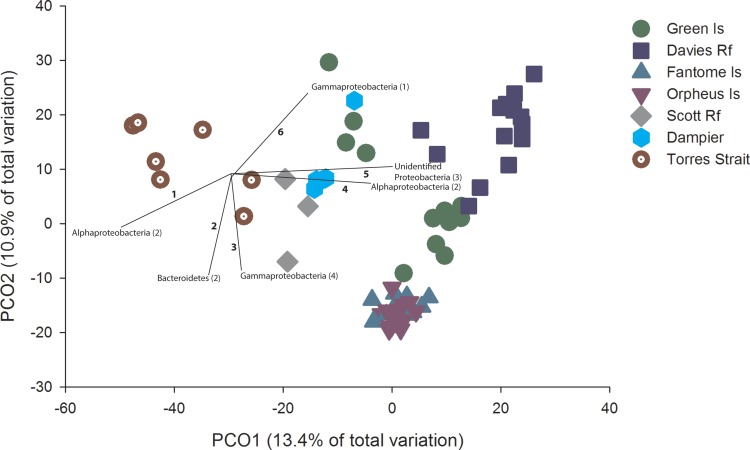
PCO with OTU vectors. PCO based on the Bray Curtis similarity of the OTUs derived from Illumina sequencing of *C. foliascens* individuals from each location. Phyla with a Spearman Rank correlation greater than 0.8 are overlaid on the plot as vectors, with the number of corresponding OTUs listed in parentheses and identified in Table S3.

PCO revealed considerable microbial variation at the OTU level according to host geography, with 24.3% of the total variation in community composition explained in the first two factors (Fig. 2). Distinct groupings within the ordination plot, based on geographic location, were evident. For example, to the right of the ordination plot there was a general grouping of GBR communities (excluding Torres St). The community composition of samples from the inshore Fantome and Orpheus Islands (separated by <10 km) were tightly grouped, with the communities at these two locations being separate from GBR communities at Green Island and Davies Reef (Fig. 2). Despite the patterns depicted in the ordination, particularly for Fantome and Orpheus Islands, the overall level of similarity (as determined by CLUSTER analysis) in microbial composition at the OTU level was 20% and all locations were significantly different (PERMANOVA, Pseudo-*F*_6,65_ = 5.57, *p* = 0.001). In fact, a significant correlation between geographic distance and community composition was identified through a comparison of the two matrices (RELATE; Rho = 0.771, *P* = 0.015). In addition, there was a significant difference in species diversity (PERMANOVA, Pseudo-*F*_6,65_ = 5.87, *p* = 0.0002) and richness (PERMANOVA, Pseudo-*F*_6,65_ = 7.20, *p* = 0.0001) estimates between locations, with Inverse Simpson values ranging from 9 to 17 and averaged estimated richness (Chao1) ranging from 3,544 to 5,751 OTUs (Table S2).

OTUs assigned to *Alphaprotobacteria*, *Bacteroidetes*, *Gammaproteobacteria* and unclassified *Proteobacteria* contributed most to the ordination, with a spearman rank correlation greater than 0.8 (Fig. 2). Notably, community differences in samples from Torres Strait were driven by a higher abundance of two *Alphaproteobacteria* OTUs (Fig. 2 and Table S2), which share 97% sequence similarity with an uncultured bacterial clone from an alkaline lake in Mexico (JN825343). Samples from communities more indicative of inshore water conditions (e.g., Fantome & Orpheus Islands) revealed higher abundances of OTU2505 and OTU109 (Fig. 2 and Table S3). Both of these *Bacteroidetes* OTUs share 100% sequence similarity to other sponge-derived microbial sequences, including a *C. foliascens* clone previously collected from Orpheus Island (Webster et al., 2013).

**Figure 3:**
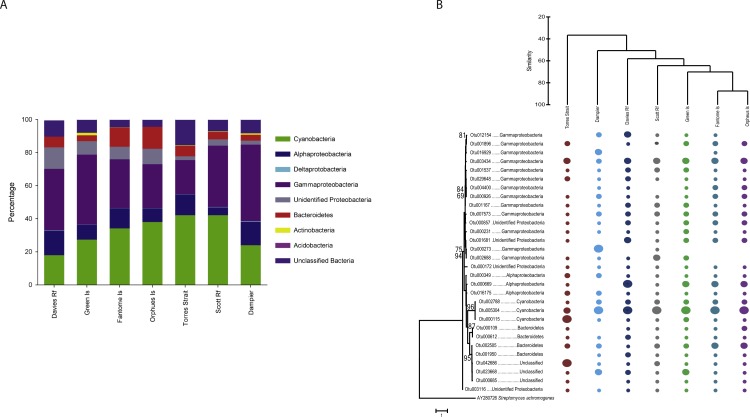
Phyla-level bar chart and OTU tree. (A) Relative abundance of each bacterial phyla, plus class for Proteobacteria, for *C. foliascens* individuals from each study location. The plot was constructed using operational taxonomic units (OTUs) that represented greater than 1% of the overall community, which accounted for 87% of the total relative abundance. (B) Relative abundance plot constructed using the 30 most abundant OTUs, with circle size corresponding to the relative abundance in each sample. Maximum likelihood tree of the OTUs (left), with bootstrap (1,000 replicates) percentages greater than 50% indicated. Samples were clustered by location using the Cluster analysis in PRIMER/PERMANOVA (top).

Taxonomic analysis revealed the community of *C. foliascens* from all locations is primarily comprised of *Gammaproteobacteria* (4,127 OTUs, relative abundance 34.2% ± 3.4%) and *Cyanobacteria* (941 OTUs, 32.2% ± 3.5%), with lower abundances of *Alphaproteobacteria*, *Bacteroidetes*, unidentified *Proteobacteria*, *Actinobacteria*, *Acidobacteria*, *Deltaproteobacteria* and unclassified bacteria also present (Fig. 3A). Although the latter phyla displayed lower relative abundances, they still comprised a high number of OTUs (Table 1). Five additional phyla were also identified; however, they comprised <1% of the overall community composition. Although different sequencing platforms preclude a direct comparison between studies, it is interesting to note that *C. foliascens* from the Red Sea hosts a similar phyla level diversity but much lower OTU level diversity than the GBR *C. foliascens* (Gao et al., 2014a). While samples possessed similar phyla-level diversity, sharing 60% similarity at the phyla-level vs. 20% at the OTU-level (percentages from CLUSTER analysis), the relative abundances of OTUs in some phyla varied between locations (Fig. 3A). For instance, samples from Fantome and Orpheus Islands have the highest relative abundance of *Bacteroidetes*, whereas *Cyanobacteria* are more abundant in samples from Torres Strait and Scott Reef (both 42%). Similar patterns of phyla-level similarity and OTU-level disparity have previously been reported for other sponge species (Simister et al., 2013; Luter, Gibb & Webster, 2014).

**Table 1:** Number of OTUs per phyla. Breakdown of the number of OTUs identified (97% similarity) per phyla, class for Proteobacteria.

	No of OTUs
Acidobacteria	25
Actinobacteria	328
Bacteroidetes	481
Cyanobacteria	931
Alphaproteobacteria	984
Deltaproteobacteria	50
Gammaproteobacteria	4,039
Unidentified Proteobacteria	1,179
Unclassified Bacteria	1,384

When considering the 30 most abundant OTUs, which comprised 68% of the total OTU abundance (Fig. S1), the phyla-level diversity decreased with only members of *Cyanobacteria*, *Gammaproteobacteria*, *Alphaproteobacteria, Bacteroidetes*, unidentified *Proteobacteria* and unclassified bacteria represented (Fig. 3B). Interestingly, together OTUs affiliated to *Gammaproteobacteria* comprised 43% of the most abundant OTUs, yet no single OTU contains a large number of reads from any location. In contrast, *Cyanobacteria* OTUs comprised only 10% of the most abundant OTUs, yet a single cyanobacterial OTU (Otu5304) dominated communities from all locations, with the exception of Torres Strait, where *Cyanobacteria* Otu115 was more abundant (Fig. 3B). Notably, all *Cyanobacteria* OTUs share high sequence similarity with clones (Otu5304 [98%] and Otu2788 [97%]: KP792324 98 & Otu115 [98%]: KJ008094) from the sponge-specific clade *Synechococcus spongiarum* (Steindler et al., 2005; Gao et al., 2014b). Clear groupings by sample location are also evident when looking at the most abundant OTUs, and consistent with the total dataset, Fantome and Orpheus Island samples share the greatest similarity (80%). A significant correlation was identified between distance matrices from the total dataset and the 30 most abundant OTUs (RELATE; Rho = 0.939, *P* = 0.001), further supporting the consistency between the two datasets.

**Figure 4:**
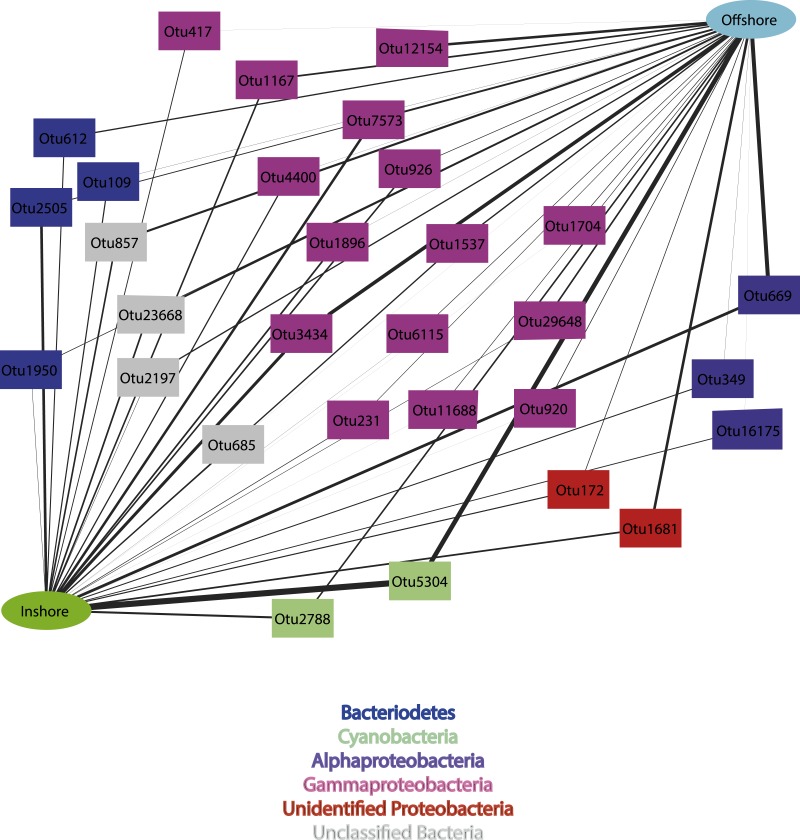
Inshore vs. offshore cytoscape network. Cytoscape network created from the 30 OTUs (97% similarity) driving the difference between Inshore and Offshore communities identified in SIMPER analysis. The width of the line represents the relative abundance of the OTU in each location.

### Inshore vs. offshore community comparison

Due to the established water quality gradient across the central GBR (Fabricius et al., 2014), comparisons between inshore and offshore locations were undertaken using samples from Eastern Australia only. Similarity percentage (SIMPER) analysis revealed the average similarity between inshore and offshore communities was 32.4%, with *Gammaproteobacteria* accounting for nearly half of the OTUs responsible for the community differences (46.6% of the overall dissimilarity) (Fig. 4). Specifically, the following OTUs were the primary drivers of dissimilarity between inshore and offshore locations (accounting for 2.65% total dissimilarity): OTU12154 (*Gammaproteobacteria*), which was completely absent from inshore samples, OTU669 (*Alphaproteobacteria*), which was more abundant in offshore samples, and OTU5304 (*Cyanobacteria*) and OTU2505 (*Bacteroidetes*), which were both more abundant in inshore samples (Fig. 4 and Table S4). Unexpectedly, amongst the top 30 OTUs driving differences observed between inshore and offshore samples, the two *Cyanobacteria* OTUs were more abundant in samples from inshore locations (Fig. 4). For instance, these OTUs comprised 88% of the total cyanobacterial relative abundance of inshore samples vs. 81% of the cyanobacterial relative abundance of offshore samples. The environmental stability of sponge microbial associations varies greatly between species and environmental conditions (Cleary et al., 2013; Cárdenas et al., 2014). For instance, the giant barrel sponge *Xestospongia muta* maintained a stable microbial community between 10–100 m (Olson & Gao, 2013), indicating a remarkable stability despite the reduction in light required for phototrophy. In contrast, in the present study we saw a 7% increase in cyanobacterial abundance in inshore samples indicating that the microbiome of *C. folisacens* is more environmentally sensitive than that of *X. muta*. Given that light is one of the most important factors influencing phototrophic sponge distributions (Wilkinson & Trott, 1985), sponges like *C*. *foliascens* are more commonly found between 0–2 m on turbid inshore reefs (Abdul Wahab et al., 2014a) compared to 10–30 m in less turbid environments on mid-shelf reefs (Wilkinson & Evans, 1989). Whilst depth differences between the two environments may also contribute to observed differences in community structure, samples collected from 10 m at the inshore Green Island site also contained higher abundances of the two *Cyanobacteria* OTUs than offshore Davies Reef samples collected at equivalent depths.

### Co-occurrence networks

**Figure 5:**
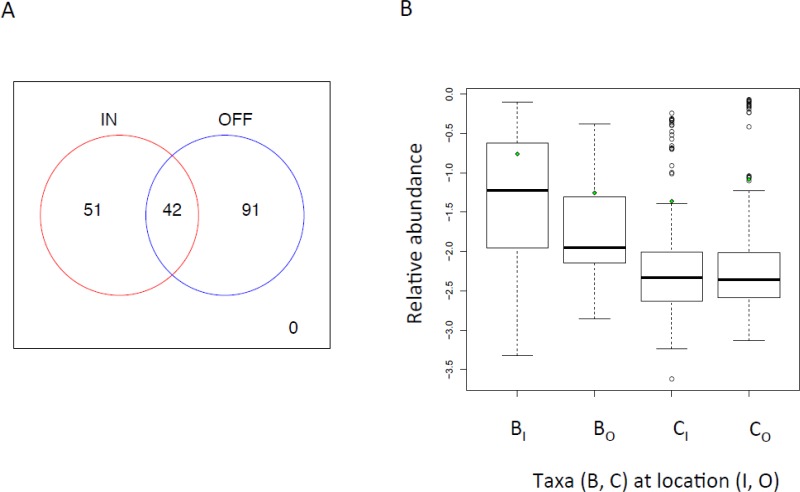
Co-occurrence outputs. (A) Venn Diagram of OTUs present in two co-occurrence networks inferred for sponge microbiome at inshore (IN) and offshore (OFF) sampling locations. The number of specialist (51, 91) and generalist taxa (42) are shown. (B) Abundance boxplot of co-occurring taxa affiliated to the phyla *Cyanobacteria* (C) and *Bacteroidetes* (B) for inshore (I) and offshore (O) locations. Mean relative abundances (log-scale) are indicated by green diamonds.

To test our hypothesis that opposing environmental conditions related to turbidity and light (based on established studies: Fabricius et al., 2014; Furnas, 2003) induce a shift in the sponge microbiome between inshore and offshore locations, we inferred two co-occurrence NWs for each of these locations and identified OTUs present and with significant co-occurrence relationships for the two habitats. We hypothesize that exclusive taxa are acclimated to these habitats. Both NWs show comparable size (*N_I_* = 93, *E_I_* = 98; *N_O_* = 133, *E_O_* = 396 where *N* = Nodes and *E* = Edges), but when comparing their fragmentation (e.g., the relative fraction of disconnected compartments within a NW) we find that the inshore NW is more fragmented (*f* = 0.61) than the offshore counterpart (*f* = 0.52). As inshore conditions are characterized by strong changes in the environment due to run-off events and variable turbidity (Fabricius et al., 2014), these results are in qualitative agreement with earlier work, showing that physical disturbance contributes to community fragmentation (Widder et al., 2014). A statistically significant co-occurrence of taxa can be motivated by specific interactions between the organisms or similar habitat preferences (Faust & Raes, 2012; Berry & Widder, 2014). Thus, by overlapping the inferred NWs we selected ecologically meaningful correlation patterns in both locations i.e., patterns that are either common or unique to each location. In Fig. 5A we depict the Venn diagram of OTUs present in the inferred NWs, where the union consists of generalists, which define OTUs that were present in both locations and specialists that represent OTUs present in either inshore or offshore locations. From the union of OTUs, we extracted the abundance of *Cyanobacteria* and *Bacteroidetes* and calculated their ratios in both environments. As expected, we found a significantly higher prevalence of *Cyanobacteria* over *Bacteroidetes* in oligotrophic, light-rich offshore locations (*C_I_*∕*B_I_* = 0.25, *C_O_*∕*B_O_* = 1.48, ANOVA, *p* < 0.0001) supporting our hypothesis that the community structure of the specialized sponge microbiome is biased by environmental conditions. In Fig. 5B we show the abundance boxplots for all OTUs affiliated to these two phyla at inshore and offshore locations. The increasing trend of *Cyanobacteria* offshore also holds if we inspect both OTUs specific for either location or OTUs prevalent in both locations. However, *Bacteroidetes* always dominate in mean relative abundance over *Cyanobacteria* irrespective of whether they were classified as specialists or generalists. *Bacteroidetes* found in the environment are known for their involvement in degrading DOM (Thomas et al., 2011) with both laboratory and *in situ* studies demonstrating the degradation of cellulose and chitin, components of DOM (Kirchman, 2002). Therefore, the highest mean relative abundances of this group in sponges collected from inshore locations, where nutrients are likely abundant, are consistent with one of the proposed physiologies for *Bacteroidetes*. However, it must be noted that not all marine *Bacteroidetes* are known to degrade particulate matter, with some utilizing proteorhodopsin to gain energy from light (Fernández-Gómez et al., 2013).

## Conclusions

Here we reveal that the ecologically important sponge *C*. *foliascens* is an important reservoir of unique microbial diversity as it harbors at least 9,401 microbial OTUs that vary significantly amongst geographic locations. Whilst high resolution sampling and environmental metadata collection are required to unequivocally define the environmental drivers of community shifts and explore temporal dynamics of the microbial communities, our finding that the ratio of *Cyanobacteria* to *Bacteroidetes* increases for sponges in oligotrophic offshore environments suggests that the composition of the *C*. *foliascens* microbiome is driven by environmental gradients including light. In addition, our analyses highlight the value of correlation approaches, such as community co-occurrence networks, for generating ecological predictions about the stability of microbe-microbe interactions under different environmental conditions.

## Supplemental Information

10.7717/peerj.1435/supp-1 Figure S1 Rank abundance plot Rank abundance plot of C. foliacens OTUs. The horizontal line represents where the 30 th OTU is placed, with the top 30 OTUs representing 68% of the accumulated proportion of abundance.

10.7717/peerj.1435/supp-2 Table S1 Sample IDs EMP sample IDs and collection location of samples from this study, including reference to samples used in the inshore/offshore comparison.

10.7717/peerj.1435/supp-3 Table S2 Alpha diversity metrics Alpha diversity metrics (average ±S.E.) of *C. foliacens* samples from each location.

10.7717/peerj.1435/supp-4 Table S3 OTU vector IDs List of OTUs contributing most to the discrimination, Spearman Rank correlation <0.8.

10.7717/peerj.1435/supp-5 Table S4 SIMPER table

10.7717/peerj.1435/supp-6 Figure S2 Co-occurrence networks Co-occurrence networks inferred from (A) 15 inshore samples and (B) 15 offshore samples. A significant co-occurrence event (edge) was placed if the SparCC correlation coefficient ≥|0.6| and *p* ≤ 0.03. Anti-correlations were removed for visualization. OTU classification denoted within the nodes.

## Additional Information and Declarations

### Field Study Permissions

The following information was supplied relating to field study approvals (i.e., approving body and any reference numbers): Permit for the study: Great Barrier Reef Marine Park Authority Permit #G12/35236.1.

### DNA Deposition

The following information was supplied regarding the deposition of DNA sequences: Processed sequences can be downloaded from the following portal: http://qiita.microbio.me/; study number 1740. In addition, sequences have been submitted to the EBI: PRJEB11983, ERP000108

### Data Availability

The following information was supplied regarding data availability: Processed sequences can be downloaded from the following portal: http://qiita.microbio.me/; study number 1740. Corresponding sample IDs can be found in Table S1.
