# Supplementary figures and images for: Biogeographic variation in the microbiome of the ecologically important sponge, Carteriospongia foliascens
Source: PeerJ. 2015 Dec 17;3:e1435. doi: 10.7717/peerj.1435 (PMC4690404; doi:10.7717/peerj.1435)

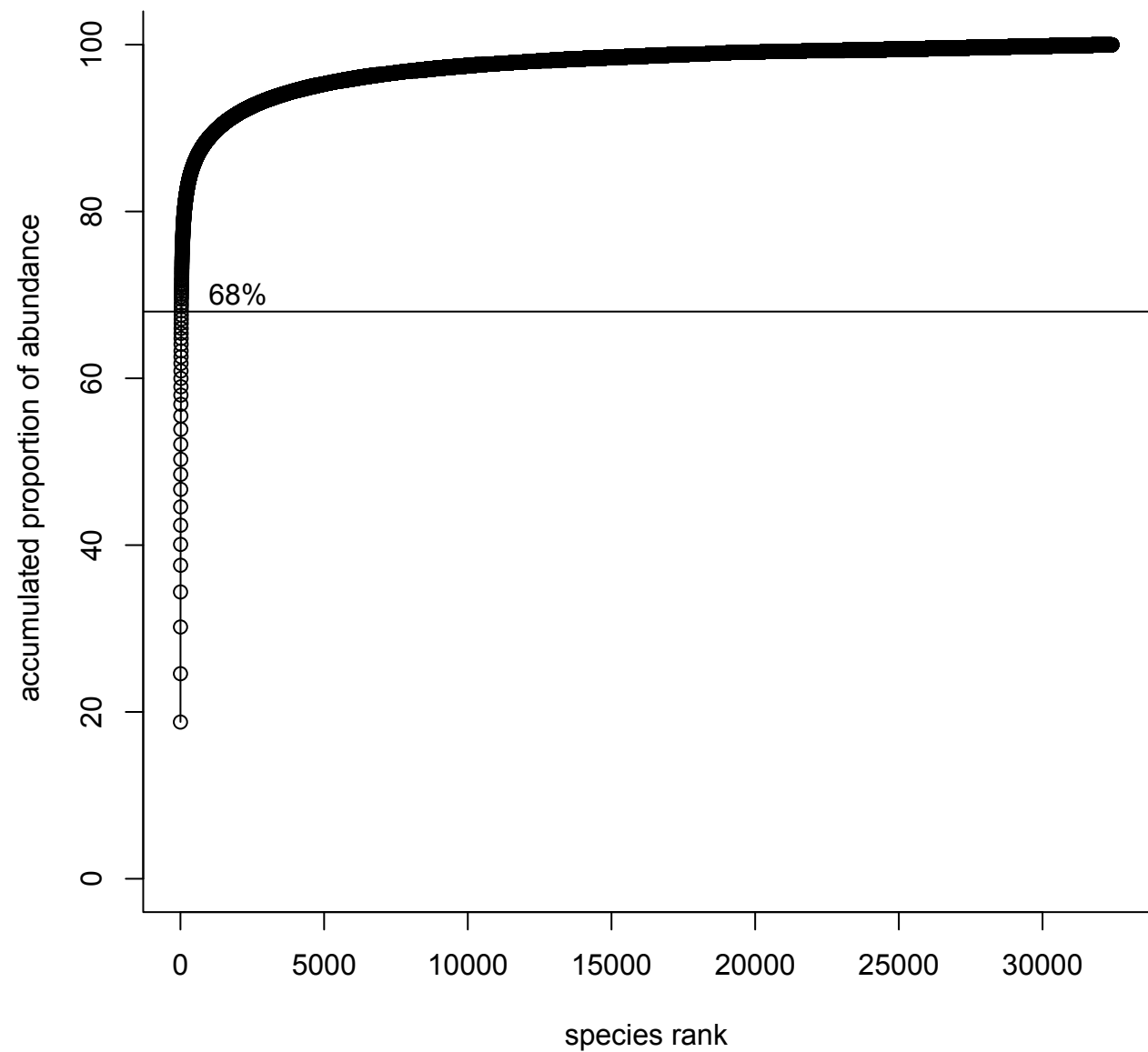

Supplement: Figure S1 — Rank abundance plot of C. foliacens OTUs. The horizontal line represents where the 30 th OTU is placed, with the top 30 OTUs representing 68% of the accumulated proportion of abundance. [file peerj-03-1435-s001.pdf]
