# Supplementary material for: Biogeographic variation in the microbiome of the ecologically important sponge, Carteriospongia foliascens
Source: PeerJ. 2015 Dec 17;3:e1435. doi: 10.7717/peerj.1435 (PMC4690404; doi:10.7717/peerj.1435)
Supplement: Table S1 — EMP sample IDs and collection location of samples from this study, including reference to samples used in the inshore/offshore comparison. [file peerj-03-1435-s002.docx]

Supplementary Table 1. EMP sample IDs, collection location and GPS coordinates of samples from this study, including reference to samples used in the inshore/offshore comparison.
