# Supplementary material for: Biogeographic variation in the microbiome of the ecologically important sponge, Carteriospongia foliascens
Source: PeerJ. 2015 Dec 17;3:e1435. doi: 10.7717/peerj.1435 (PMC4690404; doi:10.7717/peerj.1435)
Supplement: Table S2 — Alpha diversity metrics (average ±S.E.) of C. foliacens samples from each location. [file peerj-03-1435-s003.docx]

Supplementary Table 2. Alpha diversity metrics (average ± S.E.) of *C.* *foliascens* samples from each location.
