# Supplementary material for: Biogeographic variation in the microbiome of the ecologically important sponge, Carteriospongia foliascens
Source: PeerJ. 2015 Dec 17;3:e1435. doi: 10.7717/peerj.1435 (PMC4690404; doi:10.7717/peerj.1435)
Supplement: Table S3 — List of OTUs contributing most to the discrimination, Spearman Rank correlation <0.8. [file peerj-03-1435-s004.docx]

Supplementary Table 3. List of OTUs contributing most to the discrimination (Spearman Rank correlation >0.8). Sequence similarity information for each OTU as determined by BLAST is also noted. Asterisks (*) indicate a sponge host.
