# Supplementary material for: Biogeographic variation in the microbiome of the ecologically important sponge, Carteriospongia foliascens
Source: PeerJ. 2015 Dec 17;3:e1435. doi: 10.7717/peerj.1435 (PMC4690404; doi:10.7717/peerj.1435)
Supplement: Table S4 [file peerj-03-1435-s005.docx]

Supp Table 4. SIMPER breakdown for the inshore and offshore sites. Only the 30 highest contributing OTUs are shown.

|  | **Average relative abundance (%)** | | **Contribution (%)** | **Taxonomic ID** |
| --- | --- | --- | --- | --- |
| **OTU** | **Inshore** | **Offshore** |  |  |
| OTU12154 | 0.04 | 1.97 | 0.76 | Gammaproteobacteria |
| OTU2505 | 2.08 | 0.66 | 0.67 | Bacteroidetes |
| OTU669 | 2.19 | 3.11 | 0.62 | Alphaproteobacteria |
| OTU5304 | 4.76 | 3.52 | 0.6 | Cyanobacteria |
| OTU23668 | 1.31 | 1.48 | 0.51 | Unclassified Bacteria |
| OTU1896 | 1.33 | 0.08 | 0.49 | Gammaproteobacteria |
| OTU926 | 1.23 | 0.48 | 0.45 | Gammaproteobacteria |
| OTU29648 | 0.35 | 1.24 | 0.39 | Gammaproteobacteria |
| OTU2197 | 0.28 | 1.04 | 0.39 | Unclassified Bacteria |
| OTU1681 | 1.34 | 2.06 | 0.38 | Unidentified Proteobacteria |
| OTU4400 | 0.99 | 0.1 | 0.37 | Gammaproteobacteria |
| OTU7573 | 1.93 | 1.47 | 0.35 | Gammaproteobacteria |
| OTU1537 | 1.03 | 1.09 | 0.33 | Gammaproteobacteria |
| OTU109 | 0.92 | 0.16 | 0.31 | Bacteroidetes |
| OTU685 | 0.81 | 0.04 | 0.3 | Unclassified Bacteria |
| OTU349 | 0.8 | 0.17 | 0.29 | Alphaproteobacteria |
| OTU3434 | 2.34 | 2.78 | 0.29 | Gammaproteobacteria |
| OTU612 | 0.82 | 1.04 | 0.27 | Bacteroidetes |
| OTU16175 | 0.68 | 0.07 | 0.25 | Alphaproteobacteria |
| OTU857 | 1.27 | 1.66 | 0.24 | Unidentified Proteobacteria |
| OTU417 | 0.66 | 0.07 | 0.24 | Gammaproteobacteria |
| OTU1950 | 0.27 | 0.55 | 0.24 | Bacteroidetes |
| OTU231 | 0.47 | 0.37 | 0.22 | Gammaproteobacteria |
| OTU920 | 0 | 0.55 | 0.22 | Gammaproteobacteria |
| OTU2788 | 1.61 | 1.19 | 0.21 | Cyanobacteria |
| OTU1704 | 0.01 | 0.51 | 0.2 | Gammaproteobacteria |
| OTU1167 | 1.21 | 1.34 | 0.19 | Gammaproteobacteria |
| OTU172 | 0.88 | 0.53 | 0.19 | Unidentified Proteobacteria |
| OTU11688 | 0.01 | 0.44 | 0.17 | Gammaproteobacteria |
| OTU6115 | 0.08 | 0.45 | 0.17 | Gammaproteobacteria |
