# Supplementary material for: Biogeographic variation in the microbiome of the ecologically important sponge, Carteriospongia foliascens
Source: PeerJ. 2015 Dec 17;3:e1435. doi: 10.7717/peerj.1435 (PMC4690404; doi:10.7717/peerj.1435)
Supplement: Figure S2 — Co-occurrence networks inferred from (A) 15 inshore samples and (B) 15 offshore samples. A significant co-occurrence event (edge) was placed if the SparCC correlation coefficient ≥|0.6| and p ≤ 0.03. Anti-correlations were removed for visualization. OTU classification denoted within the nodes. [file peerj-03-1435-s006.pdf]

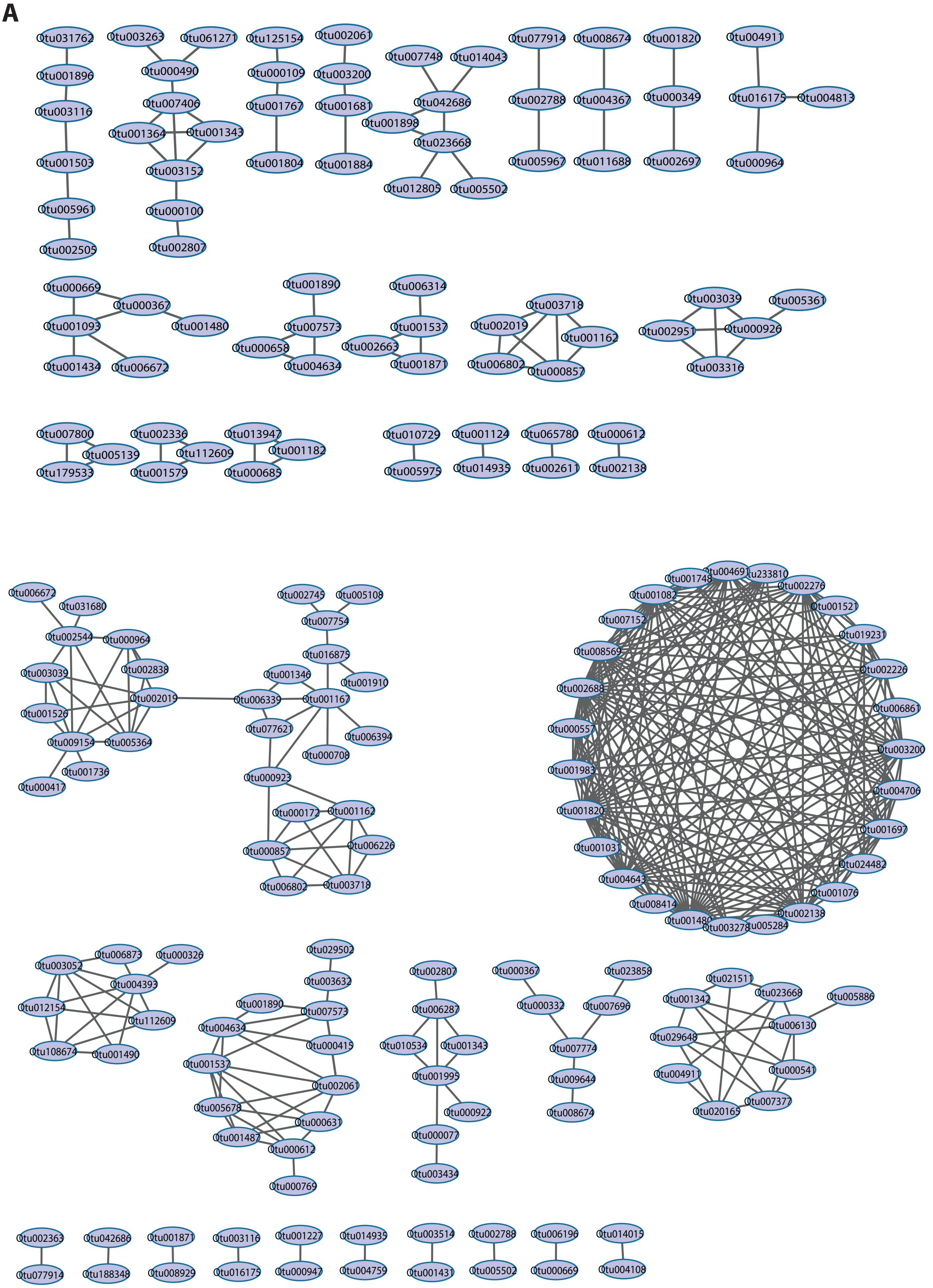

Supp Figure 2. Co-occurrence networks inferred from (A) 15 inshore samples and (B) 15 offshore samples. A significant co-occurrence event (edge) was placed if the SparCC correlation coefficient >=|0.6| and p<= 0.03. Anti-correlations were removed for visualization. OTU classification denoted within the nodes.
